# British Pharmaceutical Conference

**Published:** 1914-07-25

**Authors:** 


### British Pharmaceutical Conference.

### THE PAPERS AND HOSPITAL PHARMACISTS.

Since the establishment of the British Pharmaceutical Conference in 1863 hospital pharmacists have been among its most prominent officers. Year after year the scientific communications which have contributed most to the progress of pharmaceutical science have in many cases recorded the results of researches conducted by the chiefs of hospital laboratories; in short, pharmacy and the Conference owe much to men like the present hon. secretaries, Mr. H. Finnemore, of Guy's, and Mr. R. R. Bennett, who until recently was chief pharmacist at University College Hospital.

### Sifting Results of Research Work.

At the fifty-first annual meeting, held at Chester this week, a good proportion of the papers communicated contained material which appeals especially to institutional workers. A reference to these will be interesting, but in the first place it will be appropriate to allude to the address delivered by the President, Mr. E. H. Farr, in which an epitome of recent work on plant products was given. Mr. Farr is among the best known of the research workers in pharmacy, and his lucid account of the results of investigations conducted during the past few years will be useful for future reference. At the conclusion of his address the President made the suggestion that a Pharmacopœial Committee, representing Medicine and Pharmacy, should be set up for the purpose of sifting the results of research work, and, further, that the funds proceeding from the sale of the Pharmacopœia should be devoted exclusively to defraying the expenses connected with its production, including the investigations required in that connection. It is hoped that it may lead to some solution of the difficulties arising out of the revision of the Pharmacopœia under existing conditions.

Dealing first with the Practice Section, the paper on "The Legal Obligations of Dispensers," by Mr. H. Wippell Gadd, naturally attracted attention. Mr. Gadd stated that there was no distinction in law in respect of skill between regularly qualified dispensers and those who are not qualified if in both cases they hold themselves out to undertake dispensing for gain. A qualified dispenser is an expert; the unqualified dispenser is putting himself in the position of an expert. A dispenser is not liable criminally for any unintentional injury resulting from a lawful act unless the failure to exercise due and proper care can be imputed to him; lack of care, however, may be shown by entrusting the actual compounding to an incompetent assistant. The paper by Mr. E. S. Peck, on "Uniformity in the Dispensing of Abnormal Prescriptions," contained a suggested code of rules which pharmacists who are not infrequently faced by more or less unintelligible prescriptions should find of no little value; the safest rule is, of course, to consult the prescriber, but, unlike hospital dispensers, pharmacists who practise in shops are often called upon to dispense prescriptions written by doctors who live in another part of the country, and in such cases it is impossible to consult the prescriber; the next best thing will be to follow the rules suggested at the Conference.

### The Prices of Ether.

Of the papers read in the Science Section the one on the "Anæsthetic Ether of Commerce," by Mr. Finnemore, is perhaps of the greatest interest from the institutional point of view. An inquiry made by the author some few years ago showed that, without exception, all the large hospitals in London used methylated ether. Moreover, statistics have been published showing that the custom of anæsthetists in their private practice is very similar, and it would appear that methylated ether has very nearly replaced the higher-priced article made from rectified spirit; in fact, it is only in very small hospitals, or in special hospitals, where very few operations are performed, that ether from rectified spirit is used. Nevertheless, ether prepared from industrial methylated spirit is not recognised by the current Pharmacopœia, and it is therefore necessary to scrutinise this article as sold in order to see how it agrees with the standard. Mr. Finnemore records the results of the examination of the products of practically all the British makers, and he finds that in the main they reach a very fair average of purity; a perusal of the paper does not suggest any need to discontinue the practice of employing a good quality of ether made from industrial methylated spirit. Mr. Finnemore also presented a communication which records the results of experiments made, in collaboration with Mr. E. Williamson, with the object of determining the limits within which strychnine may be dispensed with alkaline substances without precipitation. It would appear to be a safe rule for prescribers who desire to administer strychnine with alkalies to prescribe the strychnine in the form of tincture of nux vomica; there is a great difference between the amount of strychnine hydrochloride that can be administered with alkalies when in solution in water and when in the form of the tincture or liquid extract of nux vomica.

### New Methods.

A paper by Mr. E. Quant on "Pepsin" suggests that the bacteriological purity of pepsin is not all that could be desired, and shows how improvements could be effected. Buyers of belladonna leaves should make a note of the fact that this drug is much adulterated; a paper contributed by Messrs. G. Stafford Allen and H. Deane describes the chief adulterants and methods for their detection. Messrs. R. R. Bennett and T. T. Cocking suggested an improved process for the manufacture of "Liquor Opii Sedativus." An improved method for the administration of that nauseous drug oil of male fern was suggested in a paper by Mr. F. W. Crossley Holland; the proposed formula is as follows: Liquid extract of male fern, 90 minims; gelatin, 56 grains; glycerin, 1 drachm; elixir of saccharin, 5 minims; oil of cinnamon, 1½ minims; water, 265 minims. Dispensers who would like to try this method should proceed in the following manner: Manipulate the gelatin, water, and glycerin on a water bath, and when solution is effected add the elixir of saccharin and stir gently; then add the liquid extract of male fern and oil of cinnamon, with continued stirring, until the whole mass is homogeneous. The mixture is then strained through fine gauze, previously damped, into a suitable container, where it will congeal in a few minutes. Generally speaking, the papers were quite up to the average standard of quality and practical value.

---

The Leicester Royal Infirmary benefits under the following wills: Mrs. Florence Mary Rayson, £1,000 on the death of her sister; £200, Mrs. Sarah Winterton; £200, Alderman Stephen Hilton, J.P.; £100, Mr. W. Ward Tailby, D.L., J.P.; £5, Mr. George Knight.

